# Angiotensin converting enzyme-gene polymorphisms in normal subjects, atopic individuals and those with anaphylaxis to venom, food and drugs

**DOI:** 10.1186/2045-7022-1-S1-O45

**Published:** 2011-08-12

**Authors:** Veronica Varney, Amena Warner, Angeli Ghosh, Alex Nicholas, Nazira Sumar

**Affiliations:** 1 St Helier Hospital, Immunology Dept, Surrey, UK; 2 St Helier Hospital, Surrey, UK

## Background

Circulating angiotensin-2 levels (A2) protect the circulation against sudden falls in blood pressure. A2 is generated by the enzymatic action of angiotensin converting enzyme (ACE) on angiotensin-1. The ACE genes have 2 allellic forms, with the presence 'I' or absence 'D' of a 287 base pair intron of the gene. The deletion 'DD' genotype is associated with high A2 generation & higher serum ACE levels, while the insertion 'I I' with lower serum ACE levels & ACE activity.

## Aim

To examine whether these 2 inherited polymorphisms have a different profile in patients with past anaphylaxis we compared this with control groups which included the measurement of serum ACE levels. Method: A total of 286 subjects were analysed. 118 had previous anaphylaxis, 119 were healthy controls, 49 were atopic without any previous anaphylaxis. DNA extracted from EDTA blood was analysed for ACE gene polymorphisms using polymerase chain reactions, followed by gel electrophoresis to identify the genotypes. Serum ACE levels were also measured (normal range 20-70 U/l).

## Results

See table 1 including p values from chi squared analysis.

**Table 1:** 

ACE genotype	D D	I D	I I	Healthy Controls	Atopics	Anaphylaxis	Cutaneous & RS	Mean Serum ACE Levels U/L±SEM	Serum ACE V's Healthy Controls
Healthy controls (HC) N=119 (%)	53 (45%)	44 (37%)	22 (18%)		P=0.142	P=0.009	P=0.053	48.9±6.2	

Atopic controls N=49 (%)	15 (31%)	26 (53%)	8 (16%)	P=0.142		P=0.425	P=0.025	47.9±3.5	P=0.866

Anaphylaxis N=118 (%)	30 (25%)	58 (50%)	30 (25%)	P=0.009	P=0.425			33.2±3.0	P=0.012

Cutaneous & Respiratory anaphylaxis	15 (56%)	12 (44%)	0 (0%)	P=0.053	P=0.025				

Airway angioedema ± CVS collapse	13* (14%)	46 (52%)	30 (34%)	P<0.001	P=0.024		P<0.001		

No statistical differences were found between 'Atopics' & HC, but significant differences in the genotype frequency were seen between subjects with anaphylaxis and HC.

Dividing the anaphylaxis group into 2 subgroups, there were significant differences between those with airway angio-oedema +/- cardiovascular collapse (AACVS) which is likely to be linked to ACE activity & bradykinin effects, & HC; but not between HC & cutaneous & mild respiratory symptoms (CRS) only, where histamine & not ACE is likely to be involved.

The AACVS & CRS subgroups were significantly different; p=0.001. In AACVS collapse was more likely to be associated with ID, O.R. 3.3 (95% CI 1.7, 6.3) or II, O.R. 3.1 (95% CI 1.4, 6.9) than DD genotypes p=0.001. Atopics showed a similar tendency towards a lower prevalence of DD but this was of borderline significance p=0.0821.

Physiological studies show the blood pressure responses & serum ACE levels are identical for the ID & II genotypes, with only a double dose of the "D" gene resulting in higher serum ACE levels & activity giving rapid vasoconstrictor responses in hypotension. In women, there is a higher prevalence of anaphylaxis compared with men. Interestingly the activity of the D gene is inhibited by oestrogen at the messenger RNA level, & offers cardiovascular protection against heart disease which is lost in the menopause when oestrogen falls. Serum ACE levels were similar in HC & Atopics p=0.0866, in keeping with the similar genotype. Anaphylaxis subjects had lower levels compared with HC (p=0.012) & atopic (p=0.0022) reflecting the difference in genotype.

## Conclusion

The results show a significant change in the genotype frequency between the HC, atopics and subjects who have suffered "anaphylaxis", particularly those with AACVS. These changes show a reduction in DD genotype & increases in ID & II , both of which are associated with lower Serum ACE levels & ↓ activity. Such findings may help to explain the cardiovascular collapse & airway angio-oedema in such cases.

